# HIPSTR: highest independent posterior subtree reconstruction in TreeAnnotator X

**DOI:** 10.1093/bioinformatics/btaf488

**Published:** 2025-09-09

**Authors:** Guy Baele, Luiz M Carvalho, Marius Brusselmans, Gytis Dudas, Xiang Ji, John T McCrone, Philippe Lemey, Marc A Suchard, Andrew Rambaut

**Affiliations:** Department of Microbiology, Immunology and Transplantation, Rega Institute, KU Leuven, Leuven, 3000, Belgium; School of Applied Mathematics, Getulio Vargas Foundation (FGV), Rio de Janeiro, 22250-900, Brazil; Department of Microbiology, Immunology and Transplantation, Rega Institute, KU Leuven, Leuven, 3000, Belgium; Institute of Biotechnology, Life Sciences Centre, Vilnius University, Vilnius, 01513, Lithuania; Department of Mathematics, School of Science & Engineering, Tulane University, New Orleans, LA, 70118, United States; Vaccine and Infectious Disease Division, Fred Hutchinson Cancer Center, Seattle, WA, 98109, United States; Department of Microbiology, Immunology and Transplantation, Rega Institute, KU Leuven, Leuven, 3000, Belgium; Department of Biostatistics, Fielding School of Public Health, University of California, Los Angeles, CA, 90095, United States; Department of Biomathematics, David Geffen School of Medicine, University of California, Los Angeles, CA, 90095, United States; Department of Human Genetics, David Geffen School of Medicine, University of California, Los Angeles, CA, 90095, United States; Institute of Ecology and Evolution, University of Edinburgh, Edinburgh, EH9 3FL, United Kingdom

## Abstract

**Summary:**

In Bayesian phylogenetic and phylodynamic studies, it is common to summarize the posterior distribution of trees with a time-calibrated summary phylogeny. While the maximum clade credibility (MCC) tree is often used for this purpose, we here show that a novel summary tree method—the highest independent posterior subtree reconstruction, or (HIPSTR)—contains consistently higher supported clades over MCC. We also provide faster computational routines for estimating both summary trees in an updated version of TreeAnnotator X, an open-source software program that summarizes the information from a sample of trees and returns many helpful statistics such as individual clade credibilities contained in the summary tree.

**Results:**

HIPSTR and MCC reconstructions on two Ebola virus and two SARS-CoV-2 datasets show that HIPSTR yields summary trees that consistently contain clades with higher support compared to MCC trees. The MCC trees regularly fail to include several clades with very high posterior probability (≥0.95) as well as a large number of clades with moderate to high posterior probability (≥50%), whereas HIPSTR—in particular its majority-rule extension MrHIPSTR—achieves near-perfect performance in this respect. HIPSTR and MrHIPSTR also exhibit favourable computational performance over MCC in TreeAnnotator X. Comparison to the recent CCD0-MAP algorithm yielded mixed results and requires a more in-depth investigation in follow-up studies.

**Availability and implementation:**

TreeAnnotator X is available as part of the BEAST X (v10.5.0) software package, available at https://github.com/beast-dev/beast-mcmc/releases, and on Zenodo (DOI: https://doi.org/10.5281/zenodo.4895234).

## 1 Introduction

Bayesian phylogenetic inference remains one of the most widely used frameworks to estimate the evolutionary relationship between a set of genetic or genomic sequences (Larget and Simon 1999; Huelsenbeck *et al.* 2001; Ronquist *et al.* 2012; Höhna *et al.* 2016; Bouckaert *et al.* 2019; Baele *et al.* 2025). One of the key outcomes of such a Bayesian analysis is a set of phylogenetic trees sampled from the model posterior. This set is then summarized into an easily disseminable and interpretable result—usually a single representative tree used as a framework to display important phylogenetic relationships and other quantities of interest such as divergence times or trait evolution. Given that this set of trees may contain (tens of) thousands of unique topologies, a range of summary tree calculation methods have been developed over the past decades. An extensive review of these methods is beyond the scope of this paper, but can be found in—e.g.—Bryant *et al.* (2003).

Current Bayesian phylogenetic software packages undertake a random walk through the space of trees, usually employing Metropolis-Hastings sampling (Metropolis *et al.* 1953; Hastings 1970) to attempt to sample trees from the posterior distribution as this space is explored. Were time and electricity unlimited, a preferred point-estimate of the phylogenetic tree would be the one most frequently visited (the maximum *a posteriori* or MAP tree). In practice, for all but trivially small datasets, these stochastic algorithms will likely never visit the same tree twice. Consequently, the approach taken is to consider constituent parts of the tree independently, reporting the frequency of individual clades (for rooted trees) or splits (for unrooted trees). Hereon we will refer to clades as BEAST (Bouckaert *et al.* 2019; Baele *et al.* 2025) is exclusively focused on rooted trees. For many phylogenetic questions, these clade frequencies can be used directly to provide support for competing hypotheses without considering the tree as a whole. Similarly, estimates of parameters of interest in the models employed (e.g. rates of evolution, substitution model parameters, or population size dynamics) are marginalized or averaged over all sampled trees.

However, in many cases, it is desirable to represent the totality of the phylogenetic information in the form of a single tree, ‘annotated’ with individual clade frequencies and averages or credible intervals of continuous parameters of the tree such as node ages. Furthermore, this tree can be used to visualize jointly-estimated results such as trait evolution or spatial spread. As such, it is essential that this ‘summary’ tree includes all of the highly supported clades.

The traditional approach to constructing a summary tree, one that long precedes the rise of Bayesian approaches, is the majority-rule consensus tree (Margush and McMorris 1981). Often employed to summarize resampling approaches such as bootstrapping (Felsenstein 1985) or jacknifing (Farris *et al.* 1996) with maximum-likelihood or maximum-parsimony phylogenetics, this is a tree constructed from a set of clades and their frequencies. The most popular version is the 50% majority consensus tree, a tree constructed such that it contains all of the clades with frequency over 50% (a strict consensus tree contains only 100% frequency clades). However, for non-trivial datasets these trees will not be fully resolved (bifurcating) as clades that do not meet the criteria for inclusion are collapsed into polytomies. As a result, these methods generally preclude appropriate presentation of time scales or reconstruction of geographic dispersal.

To address these limitations for analyses, where the phylogeny itself is not exclusively the result-of-interest, the BEAST packages (Bouckaert *et al.* 2019; Baele *et al.* 2025) took the approach of finding the maximum clade credibility (MCC) tree to use as the single tree representative of those sampled. The MCC tree is that amongst the sampled set which has the highest product of all the individual clade frequencies. Thus, it is a tree that the Markov chain actually visited although, in practice, only a small sample of trees is evaluated. For example, by default, BEAST stores a sample of 10 000 trees regardless of the length of the chain. This ‘thinning’ or downsampling is done to remove the autocorrelation that exists between adjacent samples and to produce a tractable set of trees in terms of both storage and the feasibility of downstream analyses. A sample of this size will likely capture all high-frequency clades but will not resolve the relative support for low-frequency clades. Furthermore, the MCC tree may be missing some clades that have less than 100% frequency if, by chance, they do not all co-occur in at least one tree of this limited sample. Sampling more frequently will not necessarily abrogate this issue because this larger set of trees will have greater autocorrelation.

We here propose a summary tree approach—the highest independent posterior subtree reconstruction (HIPSTR) algorithm—that attempts to address the limitations of both majority-rule consensus trees and MCC trees. HIPSTR aims to construct a tree that contains all the highest frequency, mutually compatible, clades even if that specific tree was never actually sampled by the MCMC. We also present MrHIPSTR, a majority-rule extension of HIPSTR, that explicitly focuses on including all clades with at least 50% frequency in its summary tree.

Since implementing the approach described here, related work has been presented by Berling *et al.* (2025) which is based on the conditional clade distribution (CCD) which offers an advanced estimate of the posterior probability distribution of the tree space. The authors extend the applicability of CCDs by introducing a new parametrization for CCDs and describing fixed-parameter tractable algorithms to compute the tree with highest probability. Of specific interest to the method we present here is the CCD0-MAP summary tree which Berling *et al.* (2025) recommend as the preferred point estimator for Bayesian phylogenetic inference of time trees. For very large trees, we found that CCD0-MAP—and to a lesser extent its faster approximation (ACCD0-MAP)—is inhibited by computational issues that may prevent the computation of the CCD0-MAP tree.

## 2 Materials and methods

### 2.1 Algorithm

**Figure 1.**
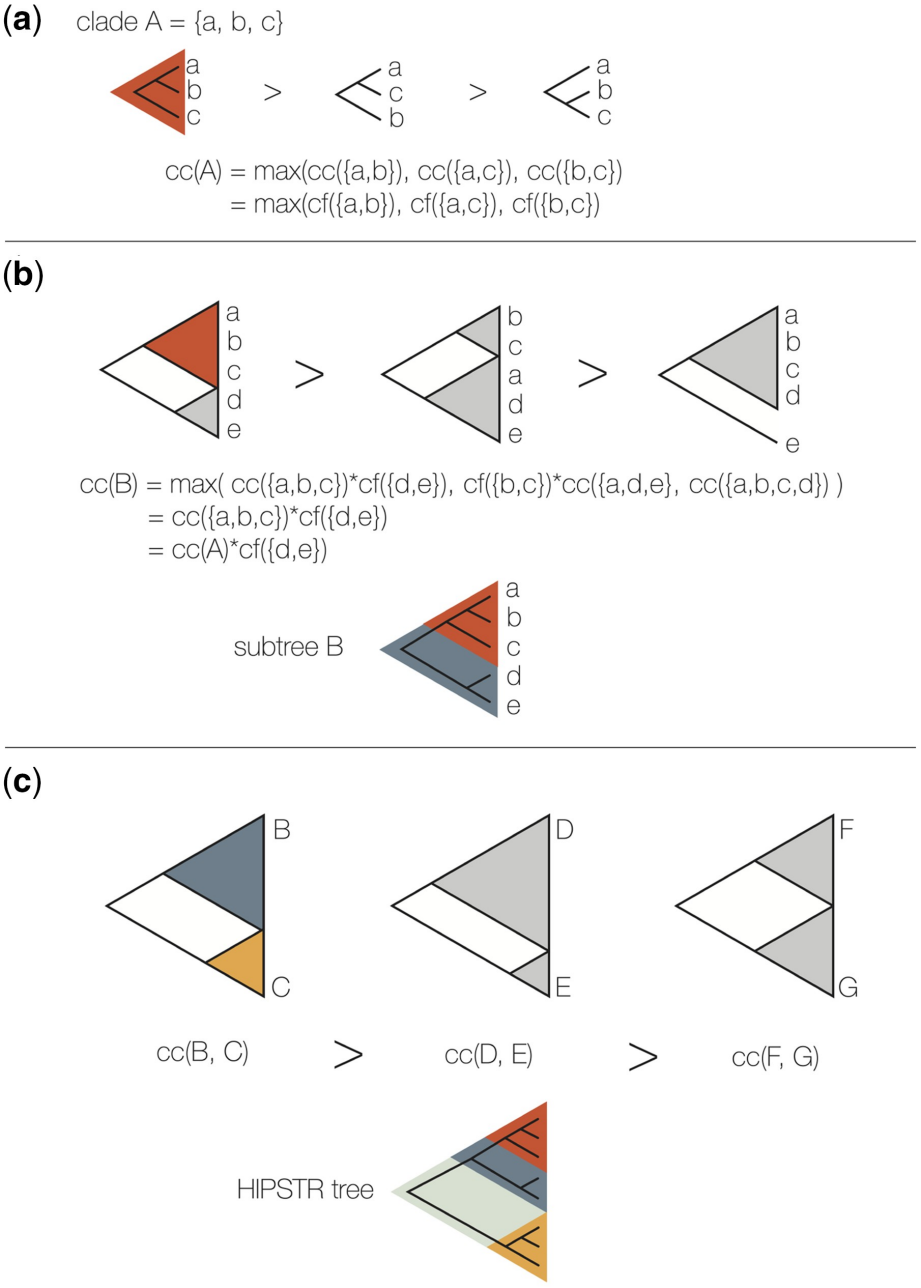
Stepwise construction of the HIPSTR summary tree. (a) For the smallest encountered clades, the MCST is simply determined by the maximum of the observed clade frequencies; (b) moving higher up in the tree towards subtrees, the MCST is the maximum product of the credibility of each pair of the subtree’s descendant clades; and (c) this process continues until we reach the root node, at which point the entire HIPSTR summary tree has been constructed (see Algorithm 1).

We describe the HIPSTR summary tree reconstruction in Algorithm 1 and Fig. 1. An initial pass of the full set of trees collates a table of all observed clades, their clade frequency and a list of all observed pairs of child clades. These clades are then processed in increasing size order and the maximum credibility subtree (MCST) is found for each. The MCST is the fully resolved subtree of clades with the highest product of clade frequencies. For clades of size 2 there is only one subtree and the credibility is the frequency of the clade. For larger clades, each observed pair of child clades is considered and the MCST for the whole clade is the pair with the highest product of their individual MCSTs. Because these child clades are smaller, their MCSTs will already have been calculated and stored in a cache, keyed by the clade, so they will not need to be recalculated. A post-order traversal of just the MCST is performed to construct the single, fully bifurcating, HIPSTR tree. Finally, a second pass of the set of posterior trees can then accumulate distributions of parameters such as node ages, evolutionary rates, and trait values for the set of clades present in the HIPSTR tree. The MrHIPSTR summary tree reconstruction only requires a single modification to Algorithm 1, in that we add a constant (e.g. $10^{10}$, the default in TreeAnnotator X for MrHIPSTR) to the subtree credibility score if the subtree has observed frequency ≥50%, to ensure all such subtrees are included in the summary tree.

Algorithm 1 **Highest independent posterior subtree reconstruction (HIPSTR)**

1: **for all** trees in the posterior sample **do**

2: **store** all observed clades in the tree and their observed pairs of child clades;

3:  **define** CF(clade) = observed clade frequency

4:  **for all** previously observed clades **do**

5:   **increment** CF(clade)

6:  **end for**

7: **end for**

8: **create** the output tree T and **initialise** its clade decomposition C(T)

9: **for all** visited subtrees in the sample **do**

10:   **define** DESC(subtree) = pair of the subtree’s descendant subtrees

11:   **define** CC(subtree) = credibility score of a subtree = maximum of CC(DESC(subtree)) × CC(subtree) over DESC(subtree); CC(subtree of size 2) = CF(clade)

12:  **Subtree Graph Traverse** starting from the smallest subtrees, compute all CC(subtree).

13:   **store** all CC(subtree) and their associated DESC(subtree)

14:   **for all** subtrees in reverse size order **do**

15:   **resolve** the subtree by picking the decomposition with the highest CC(subtree)

16:   **add** the chosen subtree to C(T)

17:  **end for**

18: **end for**

19: **return**  T      ▹ a fully-resolved HIPSTR tree.

### 2.2 Data

We assess the performance of HIPSTR on four complete-genome datasets: two Ebola virus (EBOV) datasets, one containing 1610 genomes from the 2013 to 2016 West African EBOV epidemic (Dudas *et al.* 2017), and another containing 516 genomes from the 2018 to 2020 Nord Kivu EBOV epidemic (Kinganda-Lusamaki *et al.* 2021); and two SARS-CoV-2 datasets, one containing 3959 genomes from across Europe (Lemey *et al.* 2021), and another containing 15 616 genomes from the United Kingdom (du Plessis *et al.* 2021). We selected these datasets because of their different dimensions (see Table 1) and because they represent key pathogens that continue to pose significant threats to public health. We performed visualizations in baltic v.0.3.0 (https://github.com/evogytis/baltic).

**Table 1.** Genomic dataset properties and posterior tree sample properties of the four datasets analysed in this study.

Dataset	Tips	Trees	Clades
EBOV	516	18 002	856 780
EBOV	1610	1000	287 521
SARS-CoV-2	3959	1000	660 494
SARS-CoV-2	15 616	500	3 539 556

Further, based on our findings (but see the Results section) and our extensive experience with the 2013–16 West African EBOV dataset (Dudas *et al.* 2017; Brusselmans *et al.* 2024), we created a simulated EBOV dataset (EBOV-Sim) based on the HIPSTR tree of the original EBOV Bayesian phylogenetic analysis (https://github.com/ebov/space-time/tree/master/Analyses/Phylogenetic). We simulated a sequence dataset of 20 000 bp under an HKY substitution model with among-site rate heterogeneity (Hasegawa *et al.* 1985; Yang 1996) and a strict clock with an evolutionary rate of 1E-3 substitutions per site per year. We then inferred 10 000 posterior trees from a 100 million iterations analysis in BEAST X (v10.5.0) (Baele *et al.* 2025) under a constant population size model, using the same models as were used to generate the simulated dataset.

### 2.3 Computational aspects

The initial implementation of HIPSTR (originally called MMCC for maximum marginal clade credibility) dates back to September 2019 (https://github.com/beast-dev/beast-mcmc/commit/1d3df0eabe2bd133617cf48e9e05eaa810c88152). Since then, we have restructured important parts of the TreeAnnotator code (part of the recent release of BEAST X—v10.5.0) in a more modular form and re-implemented parts of the calculations, aimed at especially benefiting summary tree construction performance for large file sizes. This implementation benefits the computational performance of both MCC and HIPSTR over the previous version of TreeAnnotator in BEAST 1.10.4 (Suchard *et al.* 2018) (data not shown).

MCC, HIPSTR, and MrHIPSTR calculations were performed in TreeAnnotator X (v10.5.0) using the default run-time settings. The CCD0-MAP, ACCD0-MAP and CCD1-MAP calculations were performed in TreeAnnotator v2.7.7 (CCD version 1.0.3). All calculations were performed on an Apple M2 Ultra 24-core processor with 192 Gb of memory.

## 3 Results

In Table 2, we show the performance comparison of HIPSTR and MrHIPSTR over MCC in terms of summary tree construction and computational cost on the four examples. Of note, phylogenetic analyses with over a thousand genomes resort to storing fewer posterior tree samples, owing to markedly increasing file sizes and ensuing post-processing issues. HIPSTR consistently yields summary trees with higher log marginal clade credibility and mean individual clade credibility over MCC trees, while doing so in a markedly shorter time compared to the MCC algorithm. Further, HIPSTR consistently includes highly supported clades, and its majority-rule extension MrHIPSTR even achieves perfect performance in this regard, whereas MCC reconstruction regularly misses out on clades with ≥95% and occasionally even ≥99% posterior probability. We note that in the 3959-taxa SARS-CoV-2 example, both the CCD0-MAP and ACCD0-MAP algorithms fail to include 16 clades with ≥50% posterior probability. MrHIPSTR by design offers the best performance of all methods tested, while only marginally decreasing the total log marginal clade credibility compared to HIPSTR. Including well-supported clades constitutes a far more important goal of summary trees than attaining the highest possible total log marginal clade credibility, as weakly supported clades are not given any consideration when interpreting the outcomes of phylogenetic and phylogeographic analyses on empirical datasets.

**Table 2.** Summary tree reconstruction and computational performance of MCC, HIPSTR, MrHIPSTR, CCD0-MAP, ACCD0-MAP and CCD1-MAP on four genomic datasets (number of sequences between brackets).^a^

Dataset	Method	med(ICC)	ICC ≥99%	ICC ≥95%	ICC ≥50%	log(MCC)	Time
EBOV	MCC	0.1555	103/103	116/116	160/177	−1355.52	116 s
(516)	CCD0-MAP	0.2784	103/103	116/116	177/177	−923.66	643 s
	ACCD0-MAP	0.2784	103/103	116/116	177/177	−939.77	243 s
	CCD1-MAP	0.1949	100/103	112/116	165/177	−1752.89	235 s
	HIPSTR	0.2751	103/103	116/116	176/177	−950.08	61 s
	MrHIPSTR	0.2784	103/103	116/116	177/177	−950.67	61 s
EBOV	MCC	0.1780	380/381	419/421	587/634	−3960.10	10 s
(1610)	CCD0-MAP	0.3130	381/381	421/421	634/634	−2864.92	64 s
	ACCD0-MAP	0.3120	381/381	421/421	634/634	−2896.87	47 s
	CCD1-MAP	0.1660	368/381	404/421	568/634	−4686.60	35 s
	HIPSTR	0.3120	381/381	421/421	633/634	−3039.60	6 s
	MrHIPSTR	0.3130	381/381	421/421	634/634	−3040.10	6 s
SARS-CoV-2	MCC	0.0730	852/852	955/959	1214/1325	−10 146.85	40 s
(3959)	CCD0-MAP	0.1975	852/852	959/959	1315/1325	−8325.58	410 s
	ACCD0-MAP	0.1975	852/852	959/959	1315/1325	−8327.63	207 s
	CCD1-MAP	0.0720	846/852	948/959	1261/1325	−11 540.45	107 s
	HIPSTR	0.1880	852/852	959/959	1305/1325	−8630.85	21 s
	MrHIPSTR	0.1950	852/852	959/959	1325/1325	−8830.48	21 s
SARS-CoV-2	MCC	0.0120	2929/2929	2992/2995	3693/3980	−57 546.68	85 s
(15 616)	CCD0-MAP	NA	NA	NA	NA	NA	>36 h
	ACCD0-MAP	0.0500	2929/2929	2995/2995	3980/3980	−50 418.37	98 m
	CCD1-MAP	0.0060	2929/2929	2994/2995	3886/3980	−58 636.38	369 s
	HIPSTR	0.0440	2929/2929	2995/2995	3980/3980	−51 352.61	52 s
	MrHIPSTR	0.0440	2929/2929	2995/2995	3980/3980	−51 352.61	52 s
EBOV-Sim	True tree	0.5239	620/620	658/658	819/819	−1762.67	79 s
(1610)	MCC	0.4728	619/620	657/658	784/819	−2610.98	150 s
	CCD0-MAP	0.5239	620/620	658/658	819/819	−1760.87	501 s
	ACCD0-MAP	0.5239	620/620	658/658	819/819	−1774.31	308 s
	CCD1-MAP	0.4726	599/620	631/658	778/819	−3543.36	311 s
	HIPSTR	0.5239	620/620	658/658	819/819	−1786.64	81 s
	MrHIPSTR	0.5239	620/620	658/658	819/819	−1786.64	81 s

a HIPSTR and MrHIPSTR consistently yield summary trees with higher log marginal clade credibility, median individual clade credibility and number of clades with ≥50% posterior probability included over MCC, and equal or higher number of clades with ≥99% and ≥95% posterior probability. HIPSTR and MrHIPSTR are also computationally more efficient than MCC, yielding up to 2× higher performance (s, seconds; h, hours). Across all datasets tested, MrHIPSTR attains near-perfect performance in terms of including clades with ≥99%, ≥95%, and ≥50% support, the key goal of summary tree construction methods. CCD0-MAP and ACCD0-MAP yield highly similar results to HIPSTR but attain higher log(MCC) values at the expense of much increased computation times. CCD1-MAP performance was far inferior to all other methods tested. log(MCC), log marginal clade credibility; med(ICC), median individual clade credibility. NA, result not available due to TreeAnnotator v2.7.7 not completing within 36 h.

On the datasets tested HIPSTR and MrHIPSTR perform up to 2× faster than MCC. With increasing numbers of genomes—millions in the case of SARS-CoV-2—being used in phylogenetic inference, computational performance for summary tree methods needs to be considered. While offering solid performance in terms of including well-supported clades, the CCD0-MAP method—and to a lesser extent the ACCD0-MAP method—struggles in this regard when it comes to very large datasets.

**Figure 2.**
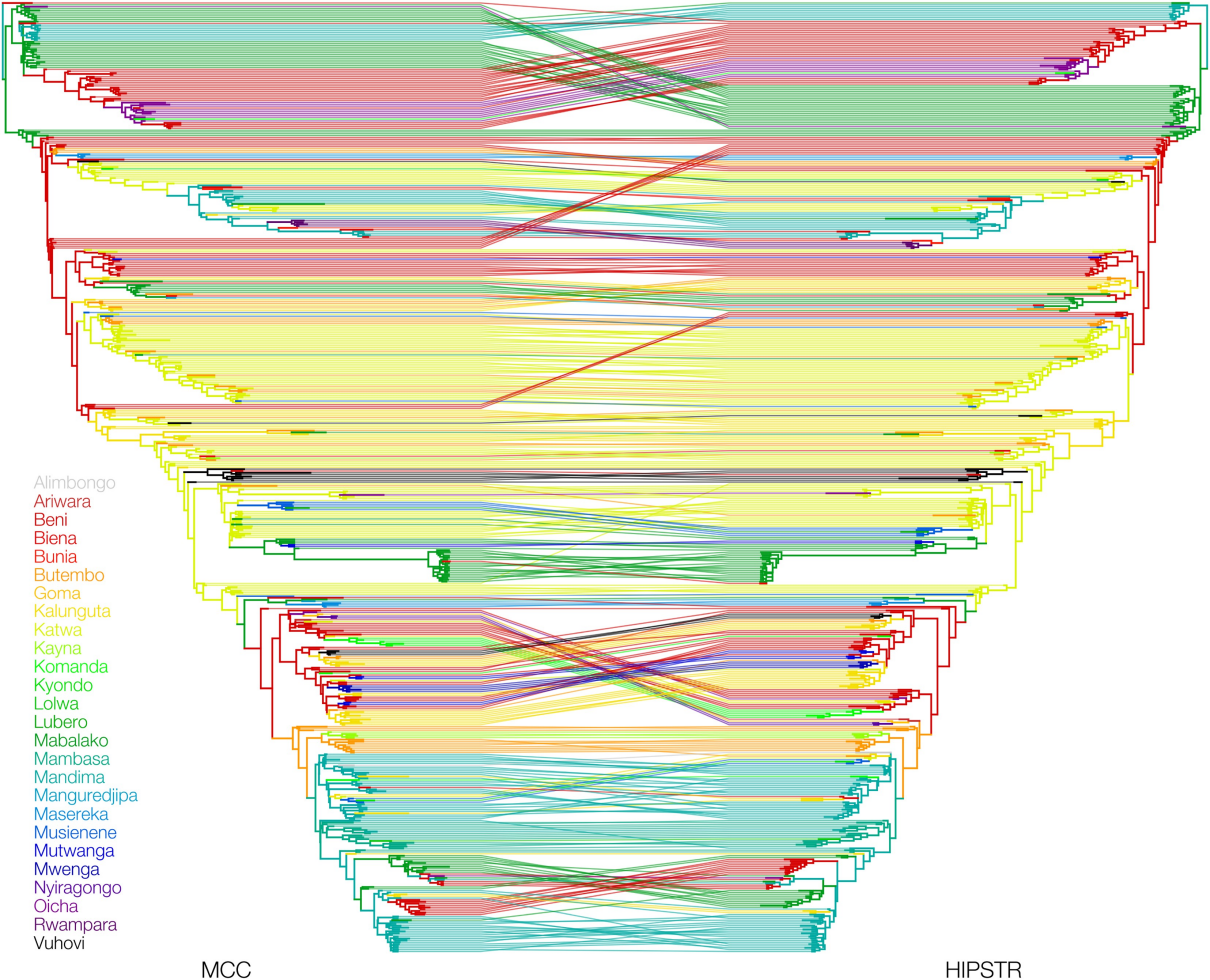
Tanglegram illustrating the similarities and differences between the MCC and HIPSTR trees for a phylogenetic analysis of the 2018–2020 Nord Kivu EBOV dataset with 516 complete genomes. The backbone of both trees is highly similar, owing to their high posterior probability (≥80%), but many differences occur between clusters with posterior probability ≥50% and with lower posterior probability (<50%; but see Figure 3). Importantly, a number of differences in terms of (number of) transitions between health zones can be observed between the MCC and HIPSTR summary trees. For example, the bottom-most clade in the MCC summary tree shows an introduction from Mandima to Beni, followed by a re-introduction from Beni to Mandima, and then back into Beni. The HIPSTR summary tree on the other hand shows a more parsimonious reconstruction of a single introduction from Mandima to Beni, showing that accurate summary tree reconstruction can have an impact on assessing the relevance of cross-border introductions of pathogens versus local transmission.

**Figure 3.**
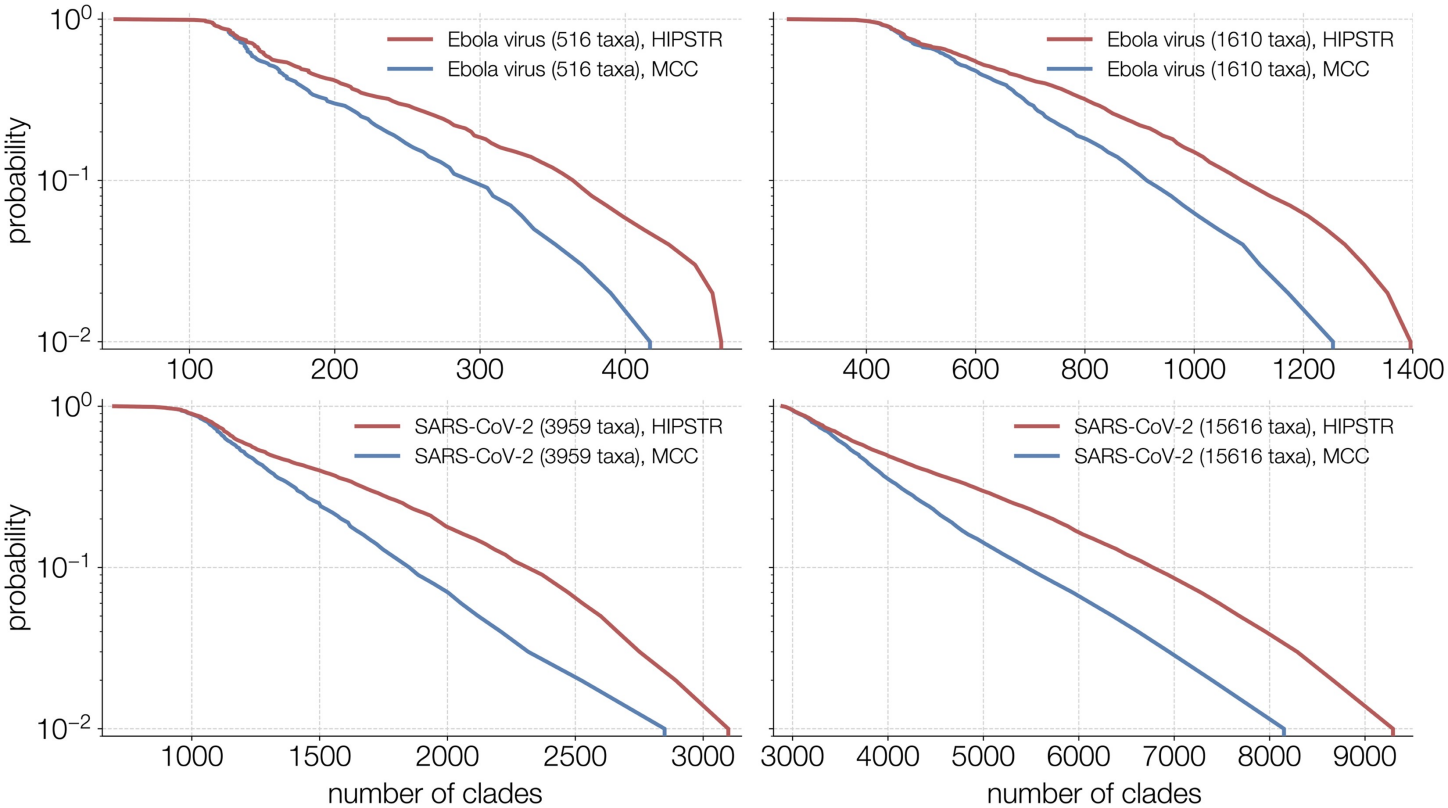
Ordered plots of the posterior probability (log scale) of all clades in the HIPSTR (red) and MCC (blue) trees. Both summary trees have all the same clades down to ∼80% posterior probability, after which the HIPSTR trees consistently include higher supported clades than the MCC trees—also evident from the differences in log marginal clade credibility; see Table 2.

Figure 2 shows a tanglegram comparing the MCC and HIPSTR summary trees for the 516-genome EBOV dataset of Kinganda-Lusamaki *et al.* (2021). We observe high similarity between both trees, especially in their backbones, due to the high posterior probability (>80%) that ensures that they become part of both trees. Away from the backbone, we also observe a large number of differences. In order to not clutter Fig. 2 with a large number of posterior probability/clade credibility values (of which summary statistics are readily available from TreeAnnotator X), we compare these values for the MCC and HIPSTR trees in more detail in Fig. 3, for all four datasets. Figure 3 shows increased divergence between MCC and HIPSTR trees as posterior support wanes, indicative of the MCC trees not including a number of relatively well-supported nodes (50%< support <80%) and the HIPSTR trees consistently selecting better supported nodes among those with lower posterior probability (<50%).

**Figure 4.**
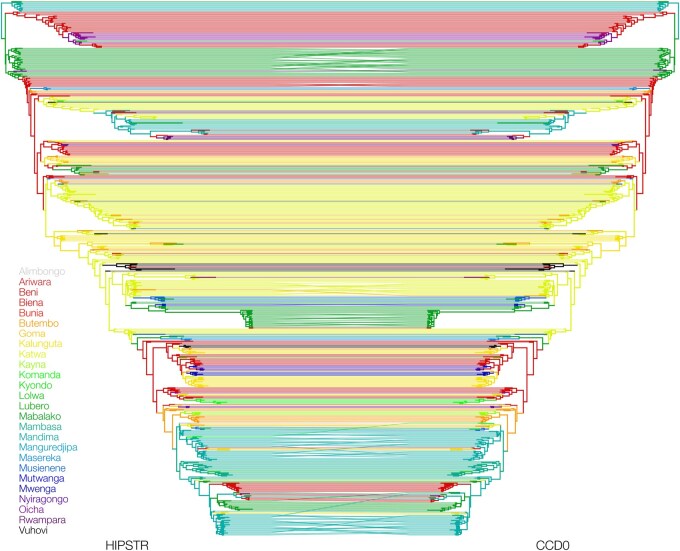
Tanglegram illustrating the similarities and differences between the HIPSTR and CCD0-MAP summary trees for a phylogenetic analysis of the 2018–2020 Nord Kivu EBOV dataset with 516 complete genomes. These highly similar summary trees have identical backbones and only differ by a single clade with ≥50% posterior probability, which is not the case for the comparison of MrHIPSTR and CCD0-MAP (see Table 2). Further, only small differences are found to be occurring in the lower-level clades.

We also showcase a comparison of the CCD0-MAP summary tree (Berling *et al.* 2025) to the MCC and HIPSTR trees for our smallest use case, i.e. the 516-genome EBOV dataset (Kinganda-Lusamaki *et al.* 2021). Figure 4 shows a tanglegram comparing the HIPSTR and CCD0-MAP summary trees for this dataset, illustrating small differences between the two and only in lower-level clades. As shown in Table 2, only a single clade with posterior probability ≥50% differs between these two summary trees, with the CCD0-MAP tree as well as MrHIPSTR (not shown) containing this clade. Note that a slight trend difference can be observed for the largest SARS-CoV-2 dataset for clade credibility values between 80% and 100%, likely owing to a difference in Bayesian inference methodology due to the much increased number of taxa (du Plessis *et al.* 2021).

## 4 Discussion

We have presented here the novel HIPSTR algorithm for reconstructing a potentially unsampled summary tree from a posterior set of phylogenetic trees, its majority-rule extension MrHIPSTR, as well as an updated version (X or v10.5.0) of TreeAnnotator. We have shown that HIPSTR summary trees contain consistently higher clade credibilities than MCC trees, on two EBOV and two SARS-CoV-2 datasets, and that computational performance of HIPSTR surpasses that of MCC reconstruction. Based on these improvements, we recommend the use of the HIPSTR and MrHIPSTR summary trees over that of the MCC tree.

A visual comparison of HIPSTR with the CCD0-MAP (employing a parametrization of a CCD based on observed clades) summary tree (Berling *et al.* 2025) on the 516-genome EBOV dataset showed very few topological differences between these methods. For the other datasets tested, the HIPSTR and CCD0-MAP algorithms yield very similar performance in terms of incorporating well-supported clades in their summary trees. The 3 959-taxa SARS-CoV-2 example stands out as being the most challenging among the datasets tested, with the CCD0-MAP approach outperforming HIPSTR but in turn being outperformed by MrHIPSTR. We have shown that MrHIPSTR offers the best performance among all the methods tested, in addition to being the fastest available summary tree method.

CCD0-MAP and to a lesser extent ACCD0-MAP can struggle with very large dataset sizes, as we have shown for our largest 15 616-taxa SARS-CoV-2 example. As an alternative, we calculated the CCD1-MAP (employing a parametrization of a CCD based on observed clade splits and thus having more parameters and requiring more uncorrelated samples) summary tree for this dataset and also provided its results for all other datasets, but these did not prove competitive with the methods proposed here (including the MCC tree for some of the datasets). Given the availability of the many other summary tree construction methods discussed here, we do not recommend using the CCD1-MAP approach.

Based on the comparisons performed in this study, we conclude that the HIPSTR and MrHIPSTR algorithms and accompanying implementations make for the most appealing choice among available time-calibrated summary phylogenetic tree approaches, taking into account their high reconstruction accuracy and very appealing computational cost. Future work on these methods will focus on assessing performance on a wider range of pathogens and dataset sizes.

## Data Availability

No new data were generated or analysed in support of this research.
